# Predictors of waterpipe smoking among university students in the Qassim region, Saudi Arabia

**DOI:** 10.18332/tid/140092

**Published:** 2021-08-30

**Authors:** Yasser S. Almogbel, Thara Aladhadh, Atheer Alammar, Atheer Aloraini, Sumayyah Alghofaili, Abdullah Almutairi, Abdulrahman AlAmri

**Affiliations:** 1 Department of Pharmacy Practice, College of Pharmacy, Qassim University, Buraidah, Saudi Arabia; 2 Saudi Pharmaceutical Industries and Medical Appliances Corporation, Buraidah, Saudi Arabia; 3 The Pharmacy Department, Specialized Medical Center Hospital, Riyadh, Saudi Arabia; 4 Department of Periodontology and Oral Medicine, College of Dentistry, Qassim University, Buraidah, Saudi Arabia; 5 Pharmaceutical Care Services, Ministry of the National Guard-Health Affairs, Riyadh, Saudi Arabia; 6 King Abdullah International Medical Research Center, Riyadh, Saudi Arabia; 7 King Saud bin Abdulaziz University for Health Sciences, Riyadh, Saudi Arabia

**Keywords:** waterpipe, cigarette smoking, university students, tobacco

## Abstract

**INTRODUCTION:**

Waterpipe smoking has gained global popularity among adolescents and young adults. This study aimed to identify the predictors of waterpipe smoking among university students in the Qassim region of Saudi Arabia.

**METHODS:**

A cross-sectional study was conducted using a pre-tested, validated, and self-administered questionnaire to identify the predictors of waterpipe smoking among university students aged >18 years in the Qassim region.

**RESULTS:**

Of the 1158 questionnaires distributed, 928 were returned with complete responses. Of these 928 participants, 820 were men (88.7%) and the majority were unmarried (95.6%). The risk of waterpipe smoking was significantly higher in students aged >26 years (OR=3.39; 95% CI: 1.30– 8.80), those who had a brother who smoked (OR=1.78; 95% CI: 1.13–2.79), and those who were married (OR=3.21; 95% CI: 1.36–7.59). Furthermore, participants who had smoked cigarettes (OR=3.18; 95% CI: 1.89–5.34) or other tobacco products (OR=6.39; 95% CI: 3.69–11.07) within the past 30 days, and students who believed that waterpipe smoking was less harmful than cigarette smoking (OR=2.61; 95% CI: 1.54–4.41) had a higher risk of engaging in waterpipe smoking. However, the risk was lower among students receiving a monthly financial aid of more than US$264 (OR=0.34; 95% CI: 0.13–0.89).

**CONCLUSIONS:**

This study revealed that higher age, being male, being married, low student financial aid, waterpipe smoker sibling, cigarette and other types of tobacco smoking in the past 30 days, and believing waterpipe smoking to be less harmful than cigarette smoking, were significant predictors of waterpipe smoking. Educational programs targeted at improving awareness of the adverse effects of waterpipe smoking should be considered for early prevention in young adults.

## INTRODUCTION

Smoking is the leading yet most preventable cause of premature death worldwide^1^. The annual premature deaths due to smoking are estimated to be 0.48 million in the US^2^. Additionally, more than 41000 deaths annually are attributed to secondhand smoking in the US^2^. The economic burden on the US population is estimated to be $300 billion annually, of which, $190 billion are related to direct medical costs^2^. An evolving tobacco trend, waterpipe smoking has gained increasing popularity worldwide, especially among adolescents and young adults^3-5^. The waterpipe is variously known as hookah, shisha, argeela, gouza, hubble-bubble, and nargeela^3^.

The term waterpipe refers to any type of instrument that involves passing tobacco smoke through water before inhalation^6^. The waterpipe includes: 1) a tobacco bowl, 2) a body made of metal, 3) a glass bowl filled with water, and 4) a rubber hose connected to a mouthpiece. The tobacco bowl is filled with tobacco and sealed with aluminum foil, and a piece of burning charcoal is placed over the foil. When the smoker inhales air through the mouthpiece, the air passes through coal, becomes hot, and burns the tobacco. Then, the smoke passes through the metal body and reaches the water glass. Finally, it travels through the hose to the mouthpiece^7^. Sweetened flavors are available as additives, giving the smoke a distinctive odor and making it fashionable among the youth^3^.

Studies have found that waterpipe smokers are exposed to more carbon monoxide than nicotine when compared to cigarette smokers^3,8,9^. In addition, waterpipe smoke contains several toxic agents such as hydrocarbons, heavy metals, and tar, which can cause cancer^3,9,10^.

Invented in India in the 16th century^11^, the waterpipe first migrated to Persia, where it evolved from its basic design to its current shape^12,13^. Later, it traveled to the Mediterranean countries^13^. Waterpipe smoking-related health risks are similar to those of cigarette smoking^14,15^. These include cardiovascular disease, cancer, pulmonary disease, higher risk of nicotine dependence, poor fetal outcome, periodontal disease, and chronic obstructive pulmonary disease^9,14-18^. Furthermore, sharing of the mouthpiece during group waterpipe smoking can cause transmission of infectious diseases^3,16^. Additionally, waterpipe smoking has negative effects on pregnancy outcomes. In particular, mothers who smoke cigarettes more than once daily are approximately two and half times more likely to deliver low birth weight babies compared to non-smoking mothers^17^.

According to the American Lung Association, waterpipe smoking is ‘an emerging deadly trend’^19^. The main source of waterpipes is the Middle East4, with Saudi Arabia alone importing more than $3.4 billion worth of tobacco products from 2010 to 2014^20^. In Saudi Arabia, waterpipe smoking is most prevalent among adolescents. However, studies on waterpipe use among university students show considerable variations in its prevalence. The cigarette smoking rate among high school students is 46.1%. While 37.4% of the students smoke only waterpipe, 16.5% smoke both waterpipe and cigarettes^21^.

A recent survey conducted in Saudi Arabia indicated that the prevalence of tobacco smoking is 12.2% among those aged ≥15 years. Daily waterpipe smokers account for 4.3% of the total population compared to 1.4% of daily cigarette, cigar, and waterpipe smokers^22^. In the past two decades, waterpipe smoking has increased among adolescents and young adults^23^, with its popularity reaching alarming levels among university students in some Arab countries. In a recent meta-analysis, three studies found the greatest prevalence of waterpipe smoking in Saudi Arabia^24^. In Saudi Arabia, two studies reported a prevalence of 21.2–40%^21,25-29^. In Lebanon, it was found to be 29%^30^. Furthermore, a study conducted in four Jordanian universities indicated that the incidence of current waterpipe smokers among university students is 30%^31^. Prevalence in other Arab countries has been reported as 5.6–19.8%^32-36^. Among non-Arab countries, in Iran the prevalence of waterpipe smoking was found to be approximately 25%^37^. In Turkey, using the Global Youth Tobacco Survey, it was reported that the prevalence of children (aged 13–15 years) who had used waterpipe was 24.6% and current waterpipe smokers were 11.2%^38^. Furthermore, the prevalence among school students in Israel was 40%^39^.

While already very popular in the Middle East, waterpipe is becoming increasingly popular in other countries as well^40^. A study conducted in the US in 2012 showed that the majority of waterpipe smokers were men aged >22 years and only 5.1% of them showed an intention to quit waterpipe smoking in the following year compared to 83.2% who had no intention of quitting. Furthermore, participants who believed that waterpipe smoking was harmful were more likely to have an intention to quit^41^.

Predicting factors associated with waterpipe smoking is important to help legislators and researchers to develop strategies to help smokers quit waterpipe smoking. A study conducted in 2012 among Saudi college students found that one-third of all male students (30.9%) were current smokers. The average age of cigarette smokers’ smoking debut was 16 years (range: 8–30). In addition, significantly heavy cigarette smokers (29.8%) had an income of approximately US$3200/year compared to non-smokers (19.3%). In general, smokers report the presence of at least one cigarette smoker in the family (48.2%) compared to non-cigarette smokers (28.4%). Cigarette smokers have one or more brothers who smoke (43.7%), and at least one cigarette smoker friend (92.9%) in contrast to non-cigarette smokers (72.3%). Additionally, students with low academic performance (grades C to D) demonstrate a two-fold risk of becoming cigarette smokers. Therefore, students with friends or family members who smoke cigarettes are more likely to begin smoking cigarettes themselves^42^. However, so far, no study has evaluated the factors associated with waterpipe smoking among Saudi college students. Thus, this study aimed to identify the predictors of waterpipe smoking among university students in Saudi Arabia.

## METHODS

### Study design and data source

A cross-sectional study was conducted using a pre-tested, validated, self-administered questionnaire to identify the predictors of waterpipe smoking among university students aged >18 years in Saudi Arabia.

This study was conducted in one of Saudi Arabia’s higher education institutions (with a strength of approximately 70000 students) in the Qassim region and the data were collected between October 2018 and April 2019 using convenience sampling. All colleges in the university, including both health science and non health science colleges, were contacted and their participation was requested. The instructors in the colleges that agreed to participate were then requested to distribute the questionnaire among the students in their lecture rooms. The questionnaire required approximately 15–20 minutes to complete. All participants had to sign an informed consent form before starting the survey. Upon completion, the students placed their questionnaires into collection boxes placed in each lecture room. The participants were informed that participation was completely voluntary and they could withdraw at any time without any negative consequences. The data were collected anonymously without any personal identifiers. The study protocol was approved by the Committee for the Protection of Human Subjects at Qassim University (Approval No.: 13518).

### Questionnaire design

The adapted questionnaire was translated from English into Arabic and then translated back into English using a back-translation technique^41,42^. Three bilingual experts validated the translated Arabic version. Moreover, the questionnaire was assessed for content and face validity by being administered to two public health specialists, who were asked to fill in the questionnaire and provide comments and feedback on its completeness and comprehensibility. The questionnaire used in this study comprised 44 questions divided into three sections as part of a larger project including objectives beyond this study (Supplementary file). However, only the questions related to the variables of interest in this study have been analyzed here (fourteen questions).

In this study, waterpipe smoking status (smoking at least once during the last 30 days [yes/no]) was the main outcome variable^41^. Based on previous studies, other variables, such as age, income (student financial aid), and sex, were included. Age was categorized into the following groups: 18–20, 21– 23, 24–26, and >26 years. Student financial aid was treated as a dichotomous variable according to the financial aid categories common in Saudi Arabia, and categorized into: <990 Saudi Riyals (SR) or US$264, and ≥990 SR. Additionally, the following variables were measured: students’ academic performance (good/poor or failed), marital status (single/married), presence of a current or former waterpipe smoker in the family (yes/no), presence of a current or former waterpipe smoker among friends (yes/no), smoking history (cigarettes or electronic cigar) within the past 30 days (yes/no), perceived level of harm compared to cigarette smoking (less harmful than cigarettes/ equally harmful/more harmful than cigarettes), and the social acceptability of waterpipe smoking among family members and friends (low/medium/high).

### Statistical analysis

Frequencies were used for the descriptive statistics. Since the predictors of waterpipe smoking were defined as dichotomous variables, chi-squared and multiple logistic regression tests were performed. Chi-squared was used as a univariate analysis to determine the association of student characteristics with quitting of waterpipe smoking. Furthermore, a probability of <0.2 for any independent variable was included in the logistic regression test of all variables. The outcomes of the statistical analyses are provided as odds ratios and presented as 95% confidence intervals. A p<0.05 was considered significant, and two-tailed tests were performed. Data were entered in MS Excel 2010, and analyzed using Stata version 16 (StataCorp LLC, College Station, Texas, US).

## RESULTS

Of the 1158 questionnaires that were distributed to students, 928 were completed and returned, resulting in a response rate of approximately 80.1%. The majority (54.9%) of the participants were aged 21–23 years and unmarried (95.6%) (Table 1). The study included 820 (88.7%) men and 105 (11.3%) women. Almost all participants (96.9%) earned ≥990 SR/month ($264/month). Approximately 94.7% of the respondents had good academic performances throughout their academic lives. Roughly 57.6% of the participants reported that they had a friend who smoked a waterpipe and 26.3% had a brother who smoked a waterpipe. Furthermore, approximately 11.6% and 5.6% of the participants had fathers and mothers who smoked, respectively, and 19.7% of the participants had smoked a cigarette at least once within the past 30 days.

**Table 1:** Demographic characteristics and bivariate analysis of predictors of waterpipe smoking among university students in the Qassim region, Saudi Arabia

*Characteristics*	*Total (n=928) ^a^*		*Non-smokers (n=743)*		*Smokers (n=185)*		*p*
	*n*	*%*	*n*	*%*	*n*	*%*	
**Age** (years)							<0.001
18–20	217	23.4	196	26.4	21	11.4	<0.001
21–23	509	54.9	405	54.6	104	56.2	
24–26	147	15.9	110	14.8	37	20.0	
>26	54	5.8	31	4.2	23	12.4	
**Sex**							0.001
Male	820	88.7	644	86.9	176	95.7	
Female	105	11.3	97	13.1	8	4.3	
**Marital status**							<0.001
Married	41	4.4	20	2.7	21	11.4	
Unmarried	881	95.6	718	97.3	163	88.6	
**Financial aid** (US$/month)							<0.001
<990 SR ($264)	29	3.1	14	1.9	15	8.1	
≥990 SR ($264)	899	96.9	729	98.1	170	91.9	
**Academic performance**							0.666
Good	874	94.7	700	94.9	174	94.0	
Poor to failed	49	5.3	38	5.1	11	6.0	
**Having a mother who smokes**							0.002
Yes	52	5.6	33	4.4	19	10.3	
No	876	94.4	710	95.6	166	89.7	
**Having a father who smokes**							0.003
Yes	108	11.6	75	10.1	33	17.8	
No	820	88.4	668	89.9	152	82.2	
**Having a brother who smokes**							<0.001
Yes	243	26.3	161	21.8	82	44.3	
No	682	73.7	579	78.2	103	55.7	
**Having a friend who smokes**							<0.001
Yes	534	57.6	404	54.4	55	29.7	
No	393	42.4	338	45.6	130	70.3	
**Smoking cigarettes in the past 30 days**							<0.001
Yes	182	19.7	79	10.7	103	56.0	
No	743	80.3	662	89.3	81	44.0	
**Using other types of tobacco in the past 30 days**							<0.001
Yes	154	16.6	56	7.6	98	53.0	
No	771	83.4	684	92.4	87	47.0	
**Perception of harm**							<0.001
Less harmful than cigarettes	128	13.8	77	10.4	51	27.7	
Equally harmful/more harmful than cigarettes	797	86.2	664	89.6	133	72.3	
**Social acceptability**							0.001
None/low	686	74.2	571	77.1	155	62.5	
Medium/high	239	25.8	170	22.9	96	37.5	

a The numbers may not total 928 for some variables due to missing values.

The results of the bivariate analysis of smoking status are summarized in Table 1. There were more smokers in the 21–23 years age group (56.2%, p<0.001). Substantially more of the waterpipe smokers were male (95.7%) than female (4.3%, p=0.001). There were more unmarried participants among the non-smoking group (97.3%) than married participants (2.7%, p=0.001). Substantially less non-waterpipe smokers (8.1%) reported receiving financial aid of >990 SR ($264) compared to non-waterpipe smokers who received financial aid of ≥990 SR (91.9%, p<0.001).

Fewer waterpipe smokers reported having a mother who smoked a waterpipe (10.3%, p=0.002), father who smoked a waterpipe (17.8%, p=0.003), and/or brother/s who smoked a waterpipe (44.3%, p<0.001). More non-waterpipe smokers than smokers reported having friends who did not smoke a waterpipe (54.4% vs 45.6%, p<0.001). Moreover, more waterpipe smokers (56.0%) had smoked cigarettes within the past 30 days compared to those who did not smoke within the past 30 days (44%, p<0.001). Additionally, of the waterpipe smokers, more had engaged in other types of smoking within the past 30 days (53.0%) than had not (47%, p<0.001). Approximately 27.7% of waterpipe smokers considered waterpipe smoking less harmful than cigarette smoking (p<0.001), whereas significantly more non-waterpipe smokers (72%) found waterpipe smoking equally or more harmful (p<0.001). Lastly, more non-waterpipe smokers (77.1%) reported less social acceptance of waterpipe smoking than waterpipe smokers (22.9%, p=0.001).

Table 2 presents the results of the multiple logistic regression analysis of the predictors of waterpipe smoking among university students in Saudi Arabia, including variables with a significance level of <0.2 in the bivariate analysis. The risk of waterpipe smoking was higher in students aged >21 years: 21–23 years (OR=3.11; 95% CI: 1.67–5.79), 24–26 years (OR=2.94; 95% CI: 1.38–6.24), and >26 years (OR=3.39; 95% CI: 1.30–8.80). Additionally, the risk of waterpipe smoking was approximately three times higher among men than in women (OR=2.73; 95% CI: 1.13–6.61), and among married individuals than in unmarried individuals (OR=3.21; 95% CI: 1.36–7.59). Furthermore, the risk of waterpipe smoking increased by 78% for individuals who had a sibling who smoked a waterpipe (OR=1.78; 95% CI: 1.13–2.79). The risk increased in waterpipe smokers who had smoked cigarettes within the past 30 days (OR=3.18; 95% CI: 1.89–5.34) and in those who had smoked other tobacco products within the past 30 days (OR=6.39; 95% CI: 3.69–11.07). Waterpipe smokers who believed that waterpipe smoking was less harmful than cigarette smoking had an increased likelihood of waterpipe smoking (OR=2.61; 95% CI: 1.54–4.41), while the risk of waterpipe smoking decreased with receiving financial aid above 990 SR ($264)/month (OR=0.34; 95% CI: 0.13–0.89).

**Table 2:** Multiple logistic regression results of predictors of waterpipe smoking among university students in the Qassim region, Saudi Arabia

*Variable*	*OR (95% CI)*	*p*
**Age** (years)		
18–20	1	
21–23	3.11 (1.67–5.79)	<0.001
24–26	2.94 (1.38–6.24)	0.005
>26	3.39 (1.30–8.80)	0.012
**Sex**		
Male	2.73 (1.13–6.61)	0.025
Female	1	
**Financial aid** (US$/month)		
<990 SR ($264)	1	
≥990 SR ($264)	0.34 (0.13–0.89)	0.028
**Marital status**		
Married	3.21 (1.36–7.59)	0.008
Unmarried	1	
**Having a mother who smokes**		
Yes	1.11 (0.46–2.68)	0.813
No	1	
**Having a father who smokes**		
Yes	0.98 (0.52–1.85)	0.975
No	1	
**Having a brother who smokes**		
Yes	1.78 (1.13–2.79)	0.012
No	1	
**Having a friend who smokes**		
Yes	0.78 (0.49–1.24)	0.306
No	1	
**Smoking cigarettes in the past 30 days**		
Yes	3.18 (1.89–5.34)	<0.001
No	1	
**Using other types of tobacco in the past 30 days**		
Yes	6.39 (3.69–11.07)	<0.001
No	1	
**Perception of harm**		
Less harmful than cigarettes	2.61 (1.54–4.41)	<0.001
Equally/more harmful than cigarettes	1	
**Social acceptability**		
None/low	1	0.518
Medium/high	1.16 (0.73–1.83)	

OR: odds ratio. CI: confidence interval.

## DISCUSSION

Waterpipe smoking is a significant public health concern. Recently, waterpipe and cigarette smoking have shown an unprecedented increase worldwide^43^.

In this study, we examined the predictors of waterpipe smoking among university students in Saudi Arabia. Among 928 students, 80% were non-waterpipe smokers and 20% were waterpipe smokers. Among the smokers, 88.7% were men and 11.3% were women. The percentage of current cigarette smokers in the past 30 days was 19.7%. A previous study conducted in the Qassim region with 371 university students identified that the inclusive predominance of waterpipe smoking (46.63%) is higher than that of cigarette smoking (26.68%). Among students, waterpipe smokers were more aware of the adverse effects, such as lower cognitive function, compared to cigarette smokers^44^.

Another study involving college students at Qassim University revealed a 40% prevalence of waterpipe smoking^29^. In 2006, a study conducted in Al-Hassa comprising 1652 secondary male students identified 30.3% of the participants as current waterpipe smokers, which is consistent with our study findings^45^. The increased prevalence of waterpipe smoking may be attributed to society’s acceptance of waterpipe smoking and the rapidly increasing number of waterpipe cafes^46^. Additionally, the most common influencing factor that increased the risk of waterpipe smoking is the misconception that waterpipe smoking is less harmful, less dangerous, and less toxic than cigarette smoking^47^. A systematic review and meta-analysis showed that the overall estimation of tobacco smoking among university students in Saudi Arabia is 17%, which is 5% higher than the mean prevalence of Saudi Arabian daily current smokers aged 15–25 years. This suggests that the national representative sample of university students in Saudi Arabia smoke at a higher rate than a marginally comparable age group^48^.

A majority of waterpipe smokers were aged 21– 23 years, and there was a remarkable increase in waterpipe smoking with increased age. This result is consistent with a similar study among Chinese adults wherein the majority of cigarettes smokers were aged 26–40 years (42.1%)^25^. The better employment status of the higher age group may affect its association with the increased risk of cigarette smoking. Moreover, in young people, cigarette smoking may be prevented by their parents who make them either stop or delay cigarette smoking^49^.

While previous studies in geographical regions similar to our study included only male students, this study included both male and female students to determine the variation in waterpipe smoking prevalence. Our study included 820 (88.7%) men and 105 (11.3%) women, which could be attributed to the gender distribution at the survey location. Male students were more likely to smoke waterpipe (95.7%) than female students who were less likely to smoke (4.3%). A previous study conducted among university students in Saudi Arabia found that a majority of waterpipe smokers were men^28^. Moreover, a previous study on 7317 adults across the 13 regions of Saudi Arabia reported that the prevalence of cigarette smoking was 32.5% among men and only 3.9% among women^50^. This may explain the low social acceptance of cigarette-smoking women in Saudi Arabian society. Furthermore, the adverse effects of smoking on women’s health are more serious, such as its effects on fertility, pregnancy, osteoporosis, and other health conditions^51^. In Saudi society, there is a community that considers female smoking of all types shameful^52^. The association between male sex and smoking waterpipe has also been observed in studies conducted outside Saudi Arabia, such as in a study of 345 Lebanese college students^30^.

Furthermore, our study revealed that receiving low financial aid was associated with an increased risk of waterpipe smoking. Students receiving financial aid below $264 were more likely to smoke waterpipe. This supports the results of a similar study conducted with 406 participants in Riyadh, in which low income was significantly associated with an increased risk of waterpipe and cigarette smoking^19^.

Education level has also been reported to play an important role in predicting waterpipe smoking; students with low education and awareness levels, such as those from underprivileged populations, have lower economic status and a higher risk of smoking waterpipe^53^. A study conducted among 600 Jordanian adults, who were cigarette smokers, reported that students with lower academic grades during their school years were more likely to smoke cigarettes^54^. However, academic performance was not significant in our study.

Being married significantly increased the likelihood of waterpipe smoking, which is consistent with a study involving Chinese cigarette smokers that showed that being married increased the risk of cigarette smoking^49^, explaining that being unmarried involves fewer social and financial responsibilities and less stress, leading to less smoking^49^.

In our study, the influence of family members (father, mother, brother) who were smokers was a significant predictor of waterpipe smoking among the students. Furthermore, the risk of waterpipe smoking with a first-degree relative who was a waterpipe smoker was found to be significant only in the bivariate analysis, except for siblings, for whom the risk was identified in both bivariate and multiple logistic regressions. Findings of a longitudinal study conducted between 1991 and 1994 on 3rd to 8th grade students in the US support the present results. The results revealed that having a family member who smoked cigarettes at home was a significant predictor of smoking at a young age^55^. Moreover, a study on 600 Jordanian adults identified the waterpipe smoking behavior of siblings as an important factor in predicting waterpipe smoking. This may occur because waterpipe smoking may become a part of the family’s social events, which may encourage the waterpipe smoking habit and affect any preventive efforts^56^.

An interesting finding in our study was that friends were not significant predictors of waterpipe smoking when compared to family members. This may be attributed to the fact that family members are strong role models who have a potential influence on young people’s waterpipe smoking behavior^42,57,58^. Future studies should focus on family members to distinguish the predictors of smoking waterpipe among university students.

This study identified that smoking cigarettes and other tobacco products within the past 30 days was a significant predictor of waterpipe smoking, whereas students who had not smoked such products in the past 30 days had a decreased risk of waterpipe smoking. A similar study conducted among university students in Saudi Arabia found that most waterpipe smokers also smoked cigarettes^59^. Another study on 744 university students in the US reported that having smoked cigarettes within the past 30 days was a strong predictor of waterpipe smoking^60^. Additionally, in a study conducted with Lebanese university students, smoking cigarettes was found to be a predictor of waterpipe smoking^30^. Another study on Jordanian adults found that participants who had smoked cigarettes within the past 30 days were 2.69 times more likely to use waterpipe^56^. This suggests that general health-risk behaviors are correlated with waterpipe smoking^61^.

The present study revealed that the majority of waterpipe smokers believed that waterpipe smoking was less harmful than cigarette smoking, which is a remarkable predictor of waterpipe smoking; this is consistent with the findings of previous studies in the US^62^ and Saudi Arabia^59^. However, in contrast to our findings, participants in a study conducted in Jordan believed that waterpipe smoking is equally or more harmful than cigarette smoking^56^. The misconception among university students that fewer risks are involved with waterpipe smoking compared to cigarettes gives them a feeling of security regarding waterpipe smoking^63^.

### Limitations

Our study had several limitations. First, as it was a cross-sectional study, causality among the variables could not be determined. Second, the generalizability of these results is limited to university student populations in Qassim region and to the small number of female participants. Third, the research was conducted using a convenience sample, non-respondents were not described, and the effect of non-participation was not recorded due to potential documentation of waterpipe smoking behavior. Furthermore, the study was conducted using self-reported questionnaires and not a direct interview; hence, the accuracy of the participants’ answers could not be confirmed and responses may have a social desirability bias. For instance, participants may not have revealed the truth, especially women, because waterpipe and other types of smoking are considered taboo, and religious beliefs prohibit all types of smoking due to their adverse health effects^52,63^. However, the anonymity of responses is expected to have mitigated this bias to some extent.

### Future perspectives

The findings of our study have several important implications. Educational programs that demonstrate the possible risks of waterpipe smoking have been reported to have an important influence on waterpipe smoking behavior^56^. Based on the predictors identified in this study, such programs should be tailored for males, higher age individuals, those receiving lower financial aid, those who are married, those who have a brother who smokes, and those who have used any form of tobacco in the past 30 days. The findings of our study support the expansion of educational programs addressing the risks of waterpipe smoking to students in all communities, especially schools, universities, and families. Furthermore, we recommend that interventions should be designed collaboratively among therapists, psychologists, and families to develop effective waterpipe smoking cessation strategies. In addition, we suggest the improvement of existing smoking cessation clinics to include waterpipe smoking and the creation of a clinic especially for female smokers within the university. Moreover, as quitting smoking early in life has been reported to increase life expectancy^64^, early interventions should be encouraged, and should focus on the prevention of waterpipe smoking in adults, especially for those whose family members are waterpipe smokers.

The government of Saudi Arabia has contributed significantly towards reducing smoking behavior through free educational programs and smoking cessation clinics. Although the smoking cessation clinic services are free, the number of smokers in Saudi Arabia remains high. Therefore, it is necessary to determine the underlying reasons behind it, particularly for smokers who do not avail themselves of the benefits of these free clinics.

Our study suggests implementation plans to counterbalance waterpipe smoking in Saudi Arabia. First, there is a need to identify the magnitude of these smoking phenomena. Second, educational policies need to be implemented. Third, policy reviews, implementation, and strengthened regulatory measures are required that would allow health policy makers to review, edit, and amend existing smoking policies in Saudi Arabia and ensure the enforcement of regulations^65^.

## CONCLUSIONS

This study examined the predictors of waterpipe smoking among university students in Saudi Arabia. We identified that the main predictors included being male, belonging to a higher age group, being married, receiving low financial aid, and having one or more brothers who smoked. Furthermore, individuals who had smoked cigarettes or used other tobacco products within the past 30 days or believed that waterpipe smoking was less harmful than cigarette smoking were significantly more likely to engage in waterpipe smoking. Awareness regarding the adverse effects and early prevention of waterpipe smoking, particularly in young adults, is crucial. Waterpipe smoking cessation at a younger age is better than treating complications of smoking later in life.

## Data Availability

The data supporting this research is available from the authors on reasonable request.
