# Supplementary material for: Predictors of waterpipe smoking among university students in the Qassim region, Saudi Arabia
Source: Tob Induc Dis. 2021 Aug 30;19:67. doi: 10.18332/tid/140092 (PMC8404266; doi:10.18332/tid/140092)
Supplement: Supplementary file 1 [file TID-19-67-s1.pdf]

Supplementary file: Questionnaire used in the study

WATERPIPE SMOKING QUESTIONNAIRE
We appreciate your help in this survey and hope you enjoy taking part in it.

Please indicate your socio-demographic information below:

1. **Age:** ________
2. **Gender:** ☐ Male ☐ Female
3. **Annual Income:**
   ☐ <$20,000 ☐ $20,000 - $35,000 ☐ $35,000 - $50,000 ☐ $50,000 - $100,000 ☐ >$100,000
4. **Please indicate your current marital status. (Select one)**
   ☐ Single ☐ Married ☐ Divorce ☐ Widowed
5. **What is your education level? (Choose one)**
   ☐ <High school degree ☐ High school degree ☐ College or University Degree ☐ Graduate degree (MS or PhD)

---

6. During the past 30 days, have you tried cigarette smoking, even one or two puffs? ☐ Yes ☐ No
7. During the past 30 days, have you tried smoking cigars, cigarillos, or little cigars, even 1 or 2 puffs? ☐ Yes ☐ No
8. During the past 30 days, have you tried smoking tobacco in a waterpipe (hookah, shisha, narghile, argila) even one or two puffs? ☐ Yes ☐ No
9. Do you believe that a governmental agency should be required to evaluate the safety of the waterpipe (hookah, shisha, narghile, and argila) before they are sold to consumers? ☐ Yes ☐ No
10. Do you believe smoking a waterpipe (hookah, shisha, narghile, and argila) is harmful to your health? ☐ Yes ☐ No
11. Compared to a regular cigarette, how harmful do you think waterpipe smoking is?
    ☐ More harmful than cigarettes ☐ As harmful as cigarettes ☐ Less harmful than cigarettes
12. What is the <u>likelihood</u> of getting addicted when using a waterpipe socially? ☐ None ☐ Low ☐ Medium ☐ High
13. What is the <u>likelihood</u> of getting addicted when using a waterpipe by oneself? ☐ None ☐ Low ☐ Medium ☐ High

---

14. What is the social acceptability of using a waterpipe among your peers? ☐ None ☐ Low ☐ Medium ☐ High
15. What is the cultural acceptability of using a waterpipe among your family members?
    ☐ None ☐ Low ☐ Medium ☐ High
16. What is the cultural acceptability of using a waterpipe among friends of your ethnicity? ☐ None ☐ Low ☐ Medium ☐ High
17. How often do you attend Middle Eastern gatherings where waterpipe are served? ☐ Daily ☐ Weekly ☐ Monthly ☐ Yearly
18. How <u>cool</u> do your peers look when they use the waterpipe? ☐ Not at all ☐ Cool ☐ Very cool
19. How old were you when you first used a waterpipe to smoke tobacco? ________ years old
20. Where were you when you first used a waterpipe to smoke tobacco?
    ☐ In a Café ☐ In my house ☐ At a family member's house ☐ At a fraternity house
    ☐ At a friend's house ☐ Dorm room ☐ Other, ________________
21. Who were you with when you first used a waterpipe to smoke tobacco? (check all that apply)
    ☐ No one, I was alone ☐ With one friend ☐ With more than one friend
    ☐ With a family member ☐ With more than one family member ☐ Other, ________

---

22. Which of the following choices best describes how often you smoke tobacco using a waterpipe?
    ☐ At least once a year but not monthly ☐ At least once a month but not weekly
    ☐ At least once a week but not daily ☐ At least once a day, or most days each month
23. When you used a waterpipe to smoke tobacco in the past 30 days, how long did a typical "waterpipe session" last? ☐ 0-30 minutes ☐ 31-60 minutes ☐ 61-90 minutes ☐ 91-120 minutes ☐ 120+ minutes
24. Do you own a waterpipe? ☐ Yes ☐ No
25. Where did you buy your waterpipe(s)? (Check all that apply)
    ☐ I do not own a waterpipe ☐ Internet ☐ Convenience store
    ☐ Tobacco shop ☐ It was a gift ☐ Street vendor ☐ Other, ________
26. Do you consider yourself "hooked" on a waterpipe? ☐ Yes ☐ No
27. When you use a waterpipe to smoke tobacco, do you usually share it with others? ☐ Yes ☐ No
28. When you use a waterpipe to smoke tobacco, is the tobacco flavored? ☐ Yes ☐ No ☐ Sometimes
29. Do you intend to quit using a waterpipe to smoke tobacco?
    ☐ Yes ☐ No
30. If yes, when are you planning to quit?
    ☐ In the next month ☐ In next 6 months ☐ In next 12 months ☐ In the future but not in the next 12 months

31. Number of times you could stop waterpipe for more than 7 days?
☐ None ☐ Once ☐ Several times ☐ It always happens

32. What percentage of income would you spend for waterpipe smoking?
☐ 1% or less of your monthly income ☐ 2%–10% of your monthly income
☐ 11%–50% of your monthly income ☐ More than 50% of your monthly income

33. Number of days you could spend without waterpipe?
☐ One day or less ☐ 2–3 days ☐ 4–7 days ☐ More than 7 days

34. Number of waterpipes you usually smoke per week?
☐ <1 waterpipe/week ☐ 1–2 waterpipes/week ☐ 3–6 waterpipes/week ☐ 7 or more waterpipes/week

35. Do you smoke waterpipe to relax your nerves?
☐ Yes, absolutely ☐ Yes, probably ☐ Yes, maybe ☐ No

36. Do you smoke waterpipe to improve your morale?
☐ Yes, absolutely ☐ Yes, probably ☐ Yes, maybe ☐ No

37. Do you smoke waterpipe when you are seriously ill?
☐ Yes, absolutely ☐ Yes, probably ☐ Yes, maybe ☐ No

38. Do you smoke waterpipe alone? ☐ Yes, always ☐ Yes, most of the times ☐ Yes, sometimes ☐ No, never

49. Are you ready not to eat in exchange for a waterpipe? ☐ Yes, absolutely ☐ Yes, probably ☐ Yes, maybe ☐ No

40. Do you smoke waterpipe for pleasure? ☐ Yes, absolutely ☐ Yes, probably ☐ Yes, maybe ☐ No

41. Do you smoke waterpipe to please others? ☐ Yes, absolutely ☐ Yes, probably ☐ Yes, maybe ☐ No

---

42. Please check your answers to the following questions by circling the appropriate number on the given scale:

1) My attempt to quit waterpipe smoking would be:

(Wise)	1	2	3	4	5	6	7	(Foolish)
(Beneficial)	1	2	3	4	5	6	7	(Harmful)
(Pleasant)	1	2	3	4	5	6	7	(Unpleasant)
(Safe)	1	2	3	4	5	6	7	(Unsafe)
(Like)	1	2	3	4	5	6	7	(Dislike)
(Good)	1	2	3	4	5	6	7	(Bad)

---

2) Quitting waterpipe smoking cessation will benefit my health.	(Likely)	1	2	3	4	5	6	7	(Unlikely)
3) Smoking waterpipe is harmful	(Agree)	1	2	3	4	5	6	7	(Disagree)
4) To quit smoking waterpipe is very important to me	(Agree)	1	2	3	4	5	6	7	(Disagree)
5) My doctor thinks I should quit smoking waterpipe	(Agree)	1	2	3	4	5	6	7	(Disagree)
6) When it comes to matters of health I want to do what my doctor thinks I should do.	(Agree)	1	2	3	4	5	6	7	(Disagree)
7) My family and friends think I should quit waterpipe smoking	(Agree)	1	2	3	4	5	6	7	(Disagree)
8) For matters of health, I want to do what my family and friends want me to do	(Agree)	1	2	3	4	5	6	7	(Disagree)
9) I am confident that I can quit waterpipe smoking	(Agree)	1	2	3	4	5	6	7	(Disagree)
10) My decision to quit smoking waterpipe is completely up to me	(Agree)	1	2	3	4	5	6	7	(Disagree)
11) I expect that I will have enough determination in the future to quit smoking	(Likely)	1	2	3	4	5	6	7	(Unlikely)
12) Having strong determination will enable me to quit waterpipe smoking	(True)	1	2	3	4	5	6	7	(False)

Thank you for your co-operation in the study. Your help is greatly appreciated.

© 2021 Almogbel Y.S. et al.
